# Focused Ultrasound Thalamotomy for Tremor Relief in Atypical Parkisnsonism

**DOI:** 10.1155/2024/6643510

**Published:** 2024-03-05

**Authors:** Alon Sinai, Maria Nassar, Lev Shornikov, Marius Constantinescu, Menashe Zaaroor, Ilana Schlesinger

**Affiliations:** ^1^Department of Neurosurgery, Rambam Health Care Campus, Haifa, Israel; ^2^Department of Neurology, Rambam Health Care Campus, Haifa, Israel; ^3^Technion Faculty of Medicine, Haifa, Israel

## Abstract

**Background:**

Magnetic resonance imaging (MRI)-guided focused ultrasound (FUS) VIM-thalamotomy has established efficacy and safety in tremor relief in patients with essential tremor and Parkinson's disease. The efficacy and safety in patients with atypical parkinsonism have not been reported.

**Objective:**

To report on the efficacy and safety of FUS VIM-thalamotomy in 8 patients with parkinsonism, multiple system atrophy-Parkinsonian type (MSA-P) (*n* = 5), and dementia with Lewy bodies (DLB) (*n* = 3).

**Methods:**

Tremor was assessed in the treated hemibody using the Clinical Rating Scale for Tremor (CRST). The motor Unified MSA Rating Scale (UMSAR) was used in the MSA-P and motor sections of the Unified Parkinson's Disease Rating Scale (UPDRS-III) in DLB patients. Cognition was measured using the Montreal Cognitive Assessment (MoCA).

**Results:**

In MSA-P and DLB patients, there was immediate tremor relief. CRST scores measured on the treated side improved compared to baseline. During the follow-up of up to 1 year tremor reduction persisted. The change in CRST scores at different time points did not reach statistical significance, probably due to the small sample size. Adverse events were transient and resolved within a year.

**Conclusions:**

In our experience, FUS VIM-thalamotomy was effective in patients with MSA-P and DLB. Larger, controlled studies are needed to verify our preliminary observations.

## 1. Introduction

Focused ultrasound (FUS) is an emerging therapy for tremor relief [1–3]. With this technique, multiple ultrasound rays are focused on a target inside the skull in order to create thermal ablation. This is a minimally invasive procedure that does not involve incisions or the implantation of foreign objects such as electrodes and a stimulator. Immediate results are seen at the end of the treatment which usually takes a few hours. The most common target for tremor relief is the ventral intermediate nucleus (VIM) of the thalamus. To date, VIM thalamotomy has shown efficacy in decreasing tremor in patients with medication-resistant tremor suffering from essential tremor (ET) [1–8]. The main side effects of treatment with FUS are gait disturbance, ataxia, paresthesia, dysgeusia, and hemiparesis [1–8]. Both short- and long-term favorable results have been reported [1–8]. Recently, the FDA approved staged bilateral FUS treatments for ET. FUS VIM-thalamotomy is also effective in tremor relief in tremor-dominant Parkinson's disease (PD) patients [8–12]. Similarly, short- and long-term beneficial effects of this treatment have been documented [8–12]. Patients with less common forms of tremor have also been treated with this technology. Two patients with dystonic tremor [13] and one with fragile X-associated tremor/ataxia syndrome (FAXTAS) [14] showed improvement in tremor. The effect of FUS thalamotomy on tremor in patients with atypical parkinsonism such as multiple system atrophy (MSA) and dementia with Lewy bodies (DLB) has not been reported.

MSA with predominant parkinsonism (MSA-P) is characterized by rigidity, akinesia/bradykinesia, postural instability, and tremor. The European MSA-Study Group found that postural tremor was present in 52% of patients and rest tremor in 36%. [15] The motor symptoms, including tremor, are generally resistant to pharmacological treatment [16]. Although some patient with clinically probable MSA may respond to levodopa, the response is typically poor or unsustained. Amantadine has also shown some efficacy, similar to levodopa [16, 17]. Deep brain stimulation in MSA-P to relieve motor symptoms showed unfavorable results and therefore is not recommended in these patients [18–20]. Thus, a large majority of MSA-P patients do not get tremor relief with the current available treatments.

Dementia with Lewy bodies is one of the most common types of degenerative dementia. In addition to progressive dementia, patients suffer from parkinsonian symptoms including bradykinesia, rigidity, and tremor. The prevalence of rest tremor and action tremor in DLB is not clear. In some studies, it was uncommon, while others reported a prevalence of 44.8% [21, 22]. Treatment of tremor and other parkinsonian symptoms in DLB is generally similar to that for PD, if somewhat less successful [23–25]. In these patients, medications are used with caution and given at smaller doses and with slower upward titration, in order not to exacerbate psychotic symptoms. In DLB patients, levodopa seems to be more effective than dopamine agonists and produces fewer side effects [26] while anticholinergic agents are generally avoided because they may worsen the cognitive state. Neurosurgical intervention with deep brain stimulation was attempted in these patients with the aim of relieving cognitive decline, not tremor, but was abandoned due to a lack of efficacy. Thus, treatment of tremor in DLB patients is currently limited.

Patients with atypical parkinsonism may be misdiagnosed at the onset of their disease as suffering from PD. Among patients that were referred to our center for FUS VIM thalamotomy, there were a few that were misdiagnosed as suffering from tremor-dominant PD, and their diagnosis was later changed to MSA-P and DLB. We report on our experience in treating these patients with atypical parkinsonism with FUS thalamotomy. To the best of our knowledge, this is the first report of FUS thalamotomy in MSA and DLB patients.

## 2. Methods

### 2.1. Patients

Among 177 patients that underwent FUS VIM-thalamotomy for tremor at Rambam Health Care Campus, Haifa, Israel, between November 2013 and January 2023, 8 were diagnosed with atypical parkinsonism including possible MSA-P (*n* = 5) and probable DLB (*n* = 3). MSA-P was diagnosed according to the Gilman and the Movement Disorder Society Criteria for the Diagnosis of Multiple System Atrophy criteria [27, 28]. DLB was diagnosed according to the revised criteria for the clinical diagnosis of DLB [29]. The diagnosis was confirmed by a movement disorder neurologist (IS or MN). The aim of FUS was to improve daily function by reducing arm tremor in the dominant hand, which could not be controlled with medication. All patients were able to provide informed consent for treatment.

Patients were examined on the day before the procedure, at the end of the procedure, and during follow-up visits at 1 month, 6 months, and 1 year.

### 2.2. Assessments

Tremor in the treated hemi-body was assessed using the Clinical Rating Scale for Tremor (hemi-CRST) (items 5-6, 8-9, 11–15; scores ranging from 0 to 36 with higher scores indicating more severe tremor) [30]. Re-emergent tremor was rated as rest tremor. The motor function unified MSA rating scale (UMSAR) [31] was used in the MSA-P and motor sections of the Unified Parkinson's Disease Rating Scale (UPDRS-III) in DLB patients. Cognition was measured using the Montreal Cognitive Assessment (MoCA).

Adverse events were documented by the neurologists after a thorough neurological examination and rated according to the Clavien-Dindo criteria (range 1 to 5; higher scores represent increased severity) [32].

This study of data collection was approved by the Rambam Health Care Campus review board. Data will be available upon request.

### 2.3. FUS Thalamotomy

In brief, patients underwent pre-procedural MRI and CT. CT images were used to assess ultrasound penetration. FUS for VIM ablation was performed using 3-T MRI and an ExAblate Neuro system (650-kHz system, Insightec LTD, Tirat Hacarmel, Israel). The procedure was performed in a staged manner in order to verify its effectiveness and avoid adverse effects. The initial brain sonication target coordinates for the VIM were calculated to be located at 25% of the AC-PC distance anterior to the PC and 14 mm lateral to the AC-PC line. When there was third ventricle enlargement, the initial target was between 14 mm lateral to the AC-PC line and 11.5 mm lateral to the third ventricle wall. The treatment was staged with a gradual increase in energy. At low temperatures of 45–50°C degree a transient or mild effect on tremor was the goal, and once achieved, the energy was gradually increased until a target temperature of 55–60°C. Treatment at the target temperature was repeated 2-3 times when possible in order to increase the probability of a long-term effect. When tremor was not totally abolished or if adverse events occurred, the target was moved according to the VIM homunculus [33]. The energy used during treatment depended on the individual's skull properties of ultrasound penetration.

### 2.4. Statistical Analysis

Hemi-CRST scores, UMSAR scores, UPDRS scores, and MoCA scores before and after the procedure were compared using Wilcoxon signed-rank tests for each time point.

## 3. Results

### 3.1. Patients

Eight patients are included in this case series (six male) with a median age of 70.0 years (range 67–84).

### 3.2. Multiple System Atrophy-Parkinsonian Type

Five patients with possible MSA-P who suffered from disabling tremors were treated with FUS. Their median age was 69 years (range 67–74) with a median disease duration of 2 years (range 1–7); two were male. Of the 5 patients, all completed the 1 month follow-up visit, 4 completed the 6 months follow-up visit, and 3 completed the 1 year follow-up visit. Due to disease progression, 2 patients did not come for follow-up visits. None died during the first year following treatment. However, 4 of 5 patients died 2–4 years after treatment. Median hemi-CRST was improved following FUS treatment from a median baseline score of 13 (*n* = 5, range 5–17) to a median score of 0 (*n* = 5, range 0-0) immediately following treatment (a statistical trend, *p*=0.06). The median CRST score remained improved at 1 month (*n* = 5, median 0, range 0–14), 6 months (*n* = 4, median 3.5, range 0–10), and 1 year (*n* = 3, median 1, range 1–4), but did not reach statistical significance see Table 1. The median UMSAR score was decreased from a median baseline score of 45 (*n* = 5, range 16–46) to a median score of 30 (*n* = 5, range 17–51) at 1 month, a median score of 23 at 6 months (*n* = 4, range 16–60), and a median score of 19 at 1 year (*n* = 3, range 17–45), although this change did not reach statistical significance.

Adverse events included transient objective and subjective gait ataxia, lip paresthesiae, and asthenia that resolved within 1–12 months (Table 1).

### 3.3. Dementia with Lewy Bodies

Three male DLB patients with disabling tremor, were treated by FUS. Their median age was 70 years (range 68–84), with a median disease duration of 14 years range [11–16]. One patient did not complete the 1 year visit due to the progression of the disease. Median hemi-CRST was improved following FUS treatment from a median baseline score of 10 (*n* = 3, range 7–23) to a median score of 0 immediately following treatment (*n* = 3, range 0-0), a median score of 0 at 1 month (*n* = 3, median 0, range 0-0), 6 months (*n* = 3, median 0, range 0-0) and 1 year (*n* = 2, median 0, range 0-0); however, this change was not statistically significant; see Table 1. The median UPDRS score at baseline was 19 (*n* = 3, range 17–27), at 1 month 17 (*n* = 3, range 9–19), at 6 months 12 (*n* = 3, range 9–20), and at 1 year 19 (*n* = 2, range 18–20). This change was not statistically significant. The median MOCA score at baseline was 21 (*n* = 3, range 19–25), at 6 months 19 (*n* = 3, range 17–24), and at 1 year 20.5 (*n* = 2, range 17–24).

Transient posttreatment gait ataxia and dysarthria that resolved within 3 months were reported in a single patient.

## 4. Discussion

In this paper, we report improvement in tremor following FUS VIM-thalamotomy in patients with possible MSA-P and probable DLB. To the best of our knowledge, this is the first report of the efficacy and safety of FUS VIM-thalamotomy in DLB and MSA-P.

The treatment was safe for all patients with mild and transient adverse effects.

Previously, FUS VIM-thalamotomy demonstrated tremor relief in PD, a synucleinopathy [8–12]. Here we report that in other synucleinopathies, MSA-P and DLB, FUS VIM thalamotomy can improve tremor as well. The favorable results could be attributed to a common pathophysiology or to a common abnormal pathway generating tremor. Since symptomatic treatment in MSA-P and DLB is limited and deep brain stimulation showed unfavorable results [18–20], our finding of long term suppression of tremor may offer patients a new treatment option. Our limited number of patients did not enable the detection of a possible effect of FUS treatment on disease progression, and this remains to be seen.

In this paper, we report on the effect of FUS on tremor. The improvement in tremor translated to improvements in both UMSAR and UPDRS scores. However, the treatment was symptomatic, and thus the disease continued to progress. Therefore, whether the improvement in tremor translates to an improvement in quality of life remains to be seen. The possibility of improving other symptoms of these disorders has not been explored; however, in PD patients, pallidotomy using FUS has shown promising results [34, 35].

It should be noted that in MSA patients, UMSAR scores were reduced over time. The reduction in UMSAR score at 1 month follow-up can be attributed to the FUS treatment. The continued reduction in scores may be explained by missing information in patients lost to follow-up due to disease progression. Thus, patients with a lower UMSAR came for follow-up visits, resulting in a pseudo-improvement of the UMSAR over time.

We report a few, mild, transient adverse events, less than in other series, probably because of our large experience with the procedure. The possibility of treatment with this technology should be carefully considered in centers where serious adverse effects are more prevalent.

The main limitation of this report is the small number of patients treated with some lost to follow-up. The number of patients at each time point was reduced over time. Thus, though improvement in tremor was evident, statistical analysis could not reach statistical significance. In some measurements, a so-called “improvement” in the scale was documented. This is probably due to the fact that patients who had a higher score were lost to follow-up maybe due to disease progression. A placebo effect cannot be ruled out. Hence, our observation should be viewed as preliminary and precludes generalization.

## 5. Conclusion

Our results offer new hope for tremor relief in MSA and DLB patients treated with unilateral FUS VIM-thalamotomy. The mild and transient adverse effects observed emphasize the safety of the procedure. Additional studies are needed to substantiate our preliminary results.

## Figures and Tables

**Table 1:** Tremor scores and adverse events following focused ultrasound thalamotomy.

Diagnosis	Age	Gender	Hemi CRST				Adverse events	
			Baseline	1 month	6 months	12 months	Description	Duration (months)
*MSA-P*								
Patient 1	69	Female	14	0	7	4	Transient gait ataxia, lip paresthesia, asthenia	3
Patient 2	67	Male	17	0	0	0		
Patient 3	74	Female	5	0	0	1	Transient gait ataxia	12
Patient 4	69	Male	13	14	10			
Patient 5	74	Female	7	5			Subjective transient gait unsteadiness	1

*DLB patients*								
Patient 1	77	Female	12	0	0	0		
Patient 2	70	Male	9	0	0	0		
Patient 3	84	Male	7	0	0	0		
Patient 4	68	Male	23	0	0		Transient gait ataxia, dysarthria	3

Hemi-CRST-clinical rating scale for Tremor in the treated hemi-body.

## Data Availability

Data are available upon reasonable request to the corresponding author.
